# Extra Hepatic Biliary Atresia associated with Choledochal Cyst: A Challenging Neonatal Obstructive Jaundice

**Published:** 2014-04-01

**Authors:** Nasrin Fatahi, Ahmad Mohammadipoor, Azin Malekmarzban

**Affiliations:** Golestan University of Medical Science, Gorgan, Iran

**Keywords:** Choledochal cyst, Biliary atresia, Jaundice, Neonate

## Abstract

Biliary atresia and choledochal cyst have the similar clinical presentation in infants. Herein, we report a case that presented with prolonged hyperbilirubinemia and abdominal distension and diagnosed as choledochal cyst. At surgery, in addition to a large choledochal cyst, biliary atresia was also encountered.

## CASE REPORT

A 22-day-old female infant was admitted due to prolonged jaundice. She was icteric from the fourth day of life along with acholic stools and abdominal distension. Direct hyperbilirubinemia was detected in blood tests. Her total bilirubin and direct bilirubin were 19mg/dl and 10.3mg/dl respectively. Clotting profile was normal. Liver function tests (LFTs) were AST (84IU/L), ALT (81IU/L), and Alkaline phosphate (2024 IU/L). The abdominal sonography showed a choledochal cyst (56x43x46mm) with few echogenic areas in it. Wall thickness and echogenicity of gallbladder as well as intra hepatic ducts were normal. Abdominal CT scan confirmed the existence of choledochal cyst (Fig. 1). The patient was operated after optimization. Operative findings included an extra hepatic choledochal cyst (type I) which was ending blind on either sides. Porta-hepatis showed type III biliary atresia. The choledochal cyst and gallbladder had no bile but mucinous secretions which were aspirated. The gallbladder and choledochal cyst were resected; the porta-hepatis dissected and Roux-en-y porto-enterostomy was performed. The wedge biopsies from porta-hepatis showed biliary atresia as donated by diffused feathery degeneration, cholestasis, pseudoacinar transformation, focal giant cell, bile duct proliferation, and fibrotic tissue. The histopathology of choledochal cyst showed a biliary epithelial lining and that of gallbladder showed congestion and near total obliteration. Postoperatively, the jaundice improved and the patient passed pigmented stool. Patient was allowed orally and discharged on short course of prednisolone, fat soluble vitamins and prophylactic antibiotic. At present, the infant is 10- months old and thriving well. The last total bilirubin is less than 1.5mg/dl. 

**Figure:**
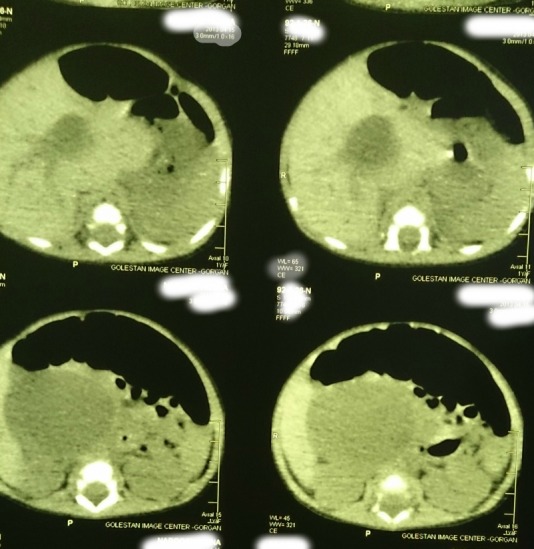
Figure 1: Cyst at porta-hepatis.

## DISCUSSION

The infantile choledochal cyst presents with obstructive jaundice, acholic stools, and hepatomegaly resembling biliary atresia and even may lead to advanced liver fibrosis.[1] In patients with biliary atresia, gallbladder is either not visualized or atretic.[2] Ultrasonographic triangular cord sign may be seen in patients with biliary atresia.[2,3] Although ultrasonography may help differentiate between both but occasionally the occurrence of both cannot be picked on it. DISIDA scan may help but as in the present case there was a cyst with patent looking gallbladder, we did not perform it. Few similar cases have been reported in literature where patient was preoperatively labelled as choledochal cyst but operation revealed biliary atresia as well. In differentiating simple choledochal cyst from choledochal cyst associated with biliary atresia, the accuracy of abdominal sonography will increase if the diameter of the extrahepatic cyst dilatation is more than 1.5 cm without an atretic gallbladder. We stress that in infantile choledochal cyst, DISIDA or HIDA scan should be added to ultrasound or CT scan that may show absent excretion of radiolabelled contrast into the duodenum thus giving a suspicion of associated biliary atresia. MRCP however is superior tool in diagnosing this concurrence.[5]

## Footnotes

**Source of Support:** Nil

**Conflict of Interest:** None

